# Coccidioidal peritonitis: A rare manifestation of disseminated coccidioidomycosis

**DOI:** 10.1016/j.idcr.2026.e02674

**Published:** 2026-07-05

**Authors:** Nicholas Cheung, Rhett Harmon, Robin L. Dietz, Shaun Chandna, Sherif Shoucri

**Affiliations:** a Department of Medicine, Olive View - UCLA Medical Center, Sylmar, CA, United States; b Department of Medicine, Ronald Reagan UCLA Medical Center, Los Angeles, CA, United States; c Department of Pathology, Department of Medicine, Olive View-UCLA Medical Center, Sylmar, CA, United States; d Division of Gastroenterology, Department of Medicine, Olive View - UCLA Medical Center, Sylmar, CA, United States; e Division of Infectious Disease, Department of Medicine, Olive View-UCLA Medical Center, Sylmar, CA, United States

**Keywords:** Peritonitis, Omental caking, Coccidioidal peritonitis, Peritoneal coccidioidomycosis, Disseminated coccidioidomycosis

## Abstract

Coccidioidomycosis (CM) is primarily a pulmonary disease but can also present with significant extrapulmonary disease. Extrapulmonary manifestations vary across organ systems, but most frequently presents as infection of the bone, brain and skin. Due to the variety of clinical presentations in disseminated disease, CM can be underrecognized, resulting in a diagnostic conundrum. We report a case of a non-immunocompromised patient initially presenting for weight loss with imaging findings mimicking peritoneal carcinomatosis, ultimately found to have CM with peritoneal involvement.

## Introduction

Coccidioidomycosis (CM) is an infection caused by *Coccidioides*, a dimorphic fungi endemic to arid, desert regions and often reported within the Western Hemisphere. CM is acquired through inhalation of airborne arthroconidia released from soil containing *Coccidioides*. Following inhalation, the organism transforms into spherules within host tissue, initiating infection. [1].

Most cases of CM manifest as self-resolving pulmonary infections due to the body’s innate immune response; however, a subset of patients may develop persistent pulmonary disease or hematogenous dissemination. Those with heavy exposure to soil such as construction workers, agricultural laborers, archaeologists, and excavators are at increased risk [2]. Other associated risk factors include immunocompromise, those with preexisting cardiopulmonary disease, African or Filipino descent, and pregnancy. [3] Disseminated disease is only estimated to manifest in ∼1% of cases, but may result in significant morbidity and mortality, such as in cases with meningitis or spinal abscess. [3] While brain, bone, and skin manifestations are the most common forms of extrapulmonary disease, dissemination to the gastrointestinal tract occurs rarely. Presentation in the abdominal cavity may result in a variety of presentations, mimicking appendicitis, cholecystitis, hepatitis, or salpingitis, while occasionally masquerading as different masses concerning for cancer. [4] We present a case of CM complicated by presumed peritoneal spread initially concerning for peritoneal carcinomatosis.

### Case report

**Fig. 1:**
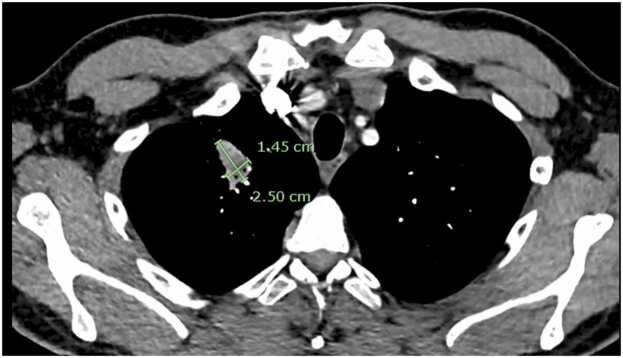
Axial Computed Tomography (CT) of the chest with a 1.45 × 2.5 cm spiculated pulmonary nodule.

A 55-year-old male smoker without significant past medical history from Southern California’s desert valley region initially presented with several months of unintentional weight loss. He underwent initial screening with computed tomography (CT) of the thorax with demonstration of two to three calcified nodules less than 3 mm in size. Three months later, he presented for further evaluation after developing worsened shortness of breath and fatigue. Labs were only significant for interval development of eosinophilia (1.0 K/cumm; ref range 0.0 – 0.4 K/cumm). CT angiography of the pulmonary arteries demonstrated a spiculated nodule in the apex of the right upper lobe, enlarged right hilar and mediastinal lymph nodes, omental edema, and scattered pulmonary micronodules (Fig. 1).

**Fig. 2:**
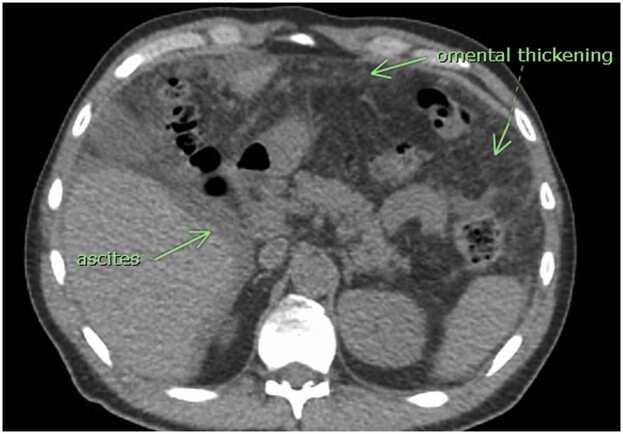
Axial CT of the abdomen demonstrating ascites and omental thickening.

Abdominal CT was subsequently ordered revealing a diffusely thickened peritoneum, trace perihepatic ascites, and omental caking (Fig. 2). Initially, there was a concern for malignancy given worsened pulmonary history and new abdominal findings; however, considering the rapid clinical progression of disease, a broad evaluation of the patient was completed, including investigations for an infectious etiology. Serologic testing for *Coccidioides* revealed elevated IgM and IgG titers and *Coccidioides* complement fixation (CF) testing was positive with a titer of 1:512, supportive of a diagnosis of disseminated CM. Due to a high degree of suspicion for presumed *Coccidioides*, the patient was started on empiric fluconazole treatment. For confirmatory testing, diagnostic paracentesis and CT-guided biopsy of abdominal lymph nodes were both attempted. However, due to interval resolution of ascites and a lack of adequate targets for tissue biopsy, both procedures were ultimately unsuccessful.

**Fig. 3:**
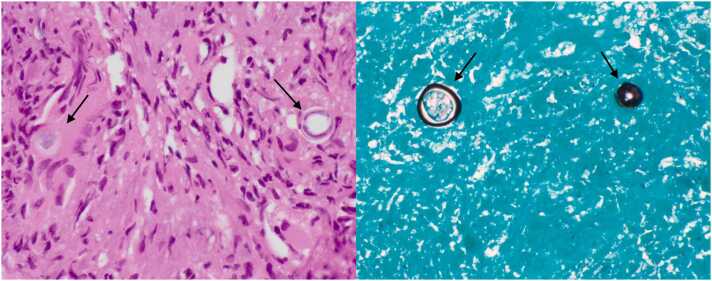
Fungal cysts seen on H&E (hematoxylin and eosin, left) and GMS (Grocott-Gomori methenamine silver, right).

The patient additionally underwent removal of a previously enlarged sebaceous cyst that had been shrinking since treatment initiation with fluconazole. Pathology results revealed granulomatous inflammation with large spherules staining positive on Grocott-Gomori Methenamine Silver (GMS) containing endospores consistent with disseminated CM (Fig. 3). Stains were negative for acid fast bacilli or malignant cells.

The patient reported symptomatic improvement on fluconazole; additionally, radiographic improvement of his pulmonary and peritoneal findings was noted. Unfortunately, the patient reported persistent headache and dizziness on fluconazole therapy. Magnetic resonance imaging of the head and a lumbar puncture returned negative, ruling out neurologic dissemination of CM. The patient was transitioned to posaconazole 300 mg with resolution of reported headache and dizziness. He continues treatment with posaconazole with complete resolution of symptoms and improvement in complement fixation from initial 1:512–1:8 on repeat testing. While formal histologic disease was unable to be confirmed in all manifestations of disease in our patient, his constellation of symptoms and robust clinical response to empiric treatment ultimately supported his presumed diagnosis of disseminated CM.

## Discussion

Peritoneal CM is an extremely rare manifestation of disseminated infection and represents a diagnostic challenge due to its nonspecific clinical presentation and radiographic similarity to intra-abdominal malignancy. Since its first description in 1939, there have only been approximately 50 cases of gastrointestinal CM reported in the literature. [5], [6] Peritoneal dissemination typically presents with nonspecific constitutional symptoms such as weight loss, fatigue, abdominal discomfort, or early satiety. Imaging findings often include ascites, diffuse peritoneal thickening, omental caking, and lymphadenopathy—features that closely resemble tuberculosis or peritoneal carcinomatosis. The differential of disease masquerading as peritoneal carcinomatosis is wide, ranging from peritoneal malignancy and lymphoproliferative disorders such as pseudomyxoma peritonei, peritoneal mesothelioma and peritoneal lymphomatosis, to benign inflammatory disease such as peritoneal leiomyomatosis or benign splenosis. [7] Infectious etiologies such as peritoneal tuberculosis, histoplasmosis, or coccidioidomycosis are also considerations. Our patient had a mixed presentation with abdominal pain and other nonspecific symptoms, making initial diagnosis a challenge, a well-reported phenomenon in cases of disseminated CM [8]. The combination of smoking history and weight loss combined with known pulmonary nodules, new CT findings of omental thickening, and ascites, all raised suspicion for malignancy with peritoneal carcinomatosis. However, the timing of disease progression, presence of eosinophilia, and primary residence in a CM-endemic region helped to prompt further evaluation for infectious etiologies, ultimately leading to the presumed diagnosis of disseminated CM.

Although definitive diagnosis of CM is ideally achieved through culture or histopathologic identification of characteristic spherules, tissue confirmation is often difficult to obtain in cases of suspected peritoneal disease due to the invasive nature of biopsy procedures and limited accessible targets. Further, it is not uncommon for the disease to be diagnosed without paracentesis, laparoscopic visualization, or biopsy. [6] While extrathoracic CM tends to have a mosaic of presentations complicating diagnosis, thorough evaluation is important to avoid unnecessary and otherwise invasive diagnostic tests and treatment. In our patient, attempts at both CT-guided biopsy and diagnostic paracentesis were unsuccessful, and histopathologic confirmation of CM was ultimately obtained incidentally from a skin lesion demonstrating classic fungal spherules. Serologic testing via enzyme immunoassay (EIA) to detect anti-coccidioidal immunoglobulin M (IgM) and immunoglobulin G (IgG) is commonly used to screen for CM, particularly when specimens for culture or histopathology are not available. [9] EIA testing is sensitive, but has been associated with false-positive results and requires confirmation with immunodiffusion and complement fixation (CF). [9] CF provides additional prognostic data as higher titer levels correlate with disease severity or dissemination. In peritoneal CM, one review of 30 cases reported a median complement fixation (CF) titer of 1:128, with declining titers correlating with treatment response; however, titers may occasionally remain normal in cases of isolated peritoneal disease. [5] Our patient had a CF titer of 1:512, suggesting dissemination of the patient’s disease to the peritoneum and soft tissue and necessitating treatment for CM.

Disseminated CM has varying degrees of morbidity and mortality. One large retrospective cohort examining untreated CM in the Veterans Affairs populace prior to the antifungal era demonstrated that cases with central nervous system involvement (CNS) held significant mortality (88%) as compared to the non-disseminated (0.65%) and non-CNS groups (25%) [10]. Within the much narrower populace of peritoneal CM, patients have typically demonstrated a robust response to antifungal treatment as well as a lack of peritonitis-associated mortality; even some untreated patients have shown good survival rate (7 out of 9 cases) [5], [6]. Previous discussions have posited these favorable outcomes to the underlying pathogenesis of peritoneal CM in addition to the excellent antifungal drug penetrance within the peritoneum compared with other sites of dissemination [5], [6].

Treatment of disseminated CM typically involves prolonged antifungal therapy. While primary pulmonary disease may be treated for a minimum of 3–6 months, chronic or extrapulmonary disease may necessitate maintenance therapy for multiple years. Further, patients with disseminated CM often require a one-to-two-year monitoring period posttreatment, due to high rates of relapse [3]. Although there is limited data guiding optimal therapy in peritoneal CM due to the rarity of the condition, treatment follows the typical management of extrapulmonary CM. Fluconazole and itraconazole remain as the initial treatments of choice due to evidence of their efficacy and historical use, although neither agent has been shown to be superior overall to the other. Fluconazole is commonly started at a dosage of 400 mg daily, increasing to 800–1200 mg in setting of treatment failure, while itraconazole is often started at 200 mg twice daily. Amphotericin B is typically reserved for treatment failure, although it may be used as an initial agent in rapidly progressing CM or in the presence of meningeal signs until symptom stabilization, before transitioning to triazole therapy [3]. While newer triazole drugs such as posaconazole and voriconazole have also had reported success in cases of treatment failure to other triazoles or amphotericin, they also pose their own risks and side effects such as hepatotoxicity. [11], [12] Isavuconazole has limited clinical data in the treatment of CM, but offers several theoretical advantages over other triazoles in *Coccidioides* treatment; namely, low minimum inhibitory concentrations, fewer drug-drug interactions, and lower adverse events, with some success demonstrated at two different CM-endemic hospitals among cases of intolerant or refractory CM. [13] Our patient initially had an improvement of symptoms on fluconazole but was transitioned to posaconazole due to medication-related side effects causing headache and dizziness, with significant clinical and serologic improvement over several months.

This case illustrates several important diagnostic considerations. First, peritoneal CM should be considered in the differential diagnosis of peritoneal carcinomatosis in patients living in or traveling from endemic regions. Second, serologic testing may provide critical diagnostic clues even when tissue confirmation is not immediately available. Finally, early recognition of disseminated CM allows timely initiation of antifungal therapy, which can significantly improve outcomes and prevent further complications such as central nervous system involvement. Although rare, peritoneal involvement represents an important manifestation of disseminated CM that may mimic malignancy and delay diagnosis. Increased awareness of this presentation among clinicians practicing in endemic regions is essential to ensure prompt recognition, appropriate diagnostic evaluation, and timely treatment.

## Ethical approval

Ethical approval was waived for this case report in accordance with institutional policy.

## CRediT authorship contribution statement

**Rhett Harmon:** Writing – original draft. **Nicholas Cheung:** Writing – review & editing, Writing – original draft. **Shaun Chandna:** Writing – original draft, Supervision. **Robin L. Dietz:** Visualization. **Sherif Shoucri:** Writing – review & editing, Writing – original draft, Supervision.

## Consent

Informed patient consent was obtained for publication of the included case details.

## Financial support

The authors received no financial support related to this work.

## Prior presentation

This case report was previously presented at the American College of Gastroenterology’s 2025 Annual Scientific Meeting on October 28, 2025, at Phoenix, Arizona.

## Declaration of Competing Interest

The authors declare that they have no known competing financial interests or personal relationships that could have appeared to influence the work reported in this paper.
